# Mesenchymal stem cells as a double-edged sword in tumor growth: focusing on MSC-derived cytokines

**DOI:** 10.1186/s11658-020-00246-5

**Published:** 2021-01-20

**Authors:** Wenqing Liang, Xiaozhen Chen, Songou Zhang, Jian Fang, Meikai Chen, Yifan Xu, Xuerong Chen

**Affiliations:** 1 Department of Orthopaedics, Zhoushan Hospital of Traditional Chinese Medicine Affiliated to Zhejiang Chinese Medical University, 355 Xinqiao Road, Dinghai District, Zhoushan, 316000 Zhejiang People’s Republic of China; 2 College of Medicine, Shaoxing University, Shaoxing, 312000 Zhejiang People’s Republic of China; 3 Department of Orthopaedics, Shaoxing People’s Hospital, The First Affiliated Hospital of Shaoxing University, Shaoxing, 312000 Zhejiang People’s Republic of China

**Keywords:** Angiogenesis, Cancer-associated fibroblasts, Immunosuppression, Metastasis, Mesenchymal stem cells

## Abstract

Mesenchymal stem cells (MSCs) show homing capacity towards tumor sites. Numerous reports indicate that they are involved in multiple tumor-promoting processes through several mechanisms, including immunosuppression; stimulation of angiogenesis; transition to cancer-associated fibroblasts; inhibition of cancer cell apoptosis; induction of epithelial–mesenchymal transition (EMT); and increase metastasis and chemoresistance. However, other studies have shown that MSCs suppress tumor growth by suppressing angiogenesis, incrementing inflammatory infiltration, apoptosis and cell cycle arrest, and inhibiting the AKT and Wnt signaling pathways. In this review, we discuss the supportive and suppressive impacts of MSCs on tumor progression and metastasis. We also discuss MSC-based therapeutic strategies for cancer based on their potential for homing to tumor sites.

## Introduction

MSCs (mesenchymal stem cells) are multipotent stem cells with high self-renewal and differentiation potential capabilities. They are a promising source for cell therapy-based regenerative medicine. The MSCs’ therapeutic features are associated with their potentials for trans-differentiation, trophic factor secretion and immunomodulation [1–3]. They already have broad applications in the treatment of degenerative diseases, including retinal degenerative disease [4, 5], multiple sclerosis [6], Alzheimer’s disease [7], Parkinson’s disease [8, 9], degenerative disc disease [10], amyotrophic lateral sclerosis [11], type 1 diabetes mellitus [12] and myocardial infarction [13].

MSCs differentiate into multiple mesenchymal cell lineages, including adipocytes, osteocytes, chondrocytes, pericytes and fibroblasts [14–19]. They can also transdifferentiate into ectodermal (neuronal) and endodermal (epithelial) lineages, and into hepatocytes under certain in vitro culture conditions [20–22]. MSCs can also participate in supporting tissue architecture and controlling inflammation [23–25].

In general, MSCs support tissue integrity as the crucial regulators of tissue homeostasis. In addition to their normal distribution in particular organs, they accumulate in damaged tissues, contributing to tissue regeneration and wound healing [24, 26, 27]. In response to tissue injury, they are mobilized from their niches upon suitable signals and recruited into tissues to participate in tissue regeneration and remodeling [28, 29]. Certain factors and conditions, including TGFβ [30], G-CSF [31] and exercise [32], can increase their mobilization and homing to peripheral blood and injured sites.

Despite intensive investigation over the years, the in vitro classification of MSCs is still unclear. Morphological observation of cultures shows a heterogeneous cell population comprising various subsets of fibroblast-like cells, round cells or flattened cells [33–35]. The differentiation and functional features of each cell subset have not been completely identified, but the minimal criteria suggested by the ISCT (International Society for Cellular Therapy) have been applied to define them. The defining characteristics are: plastic-adherent properties in standard culture circumstances; over 95% of cells in a considered population expressing CD73 and CD105, with no expression (less than 2% positive) of CD34, CD45, CD14 or CD79α, CD11b, CD19 as well as HLA-DR markers; differentiation into adipocytes, chondroblasts and osteoblasts under standard circumstances in vitro [36]. Studies indicate that MSC populations’ heterogeneity probably explains their capability of differentiating into various distinct cell types [37–39].

MSCs secrete soluble factors and mediators, such as indoleamine 2,3-dioxygenase (IDO), nitric oxide (NO), prostaglandin (PGE2), IL-10, IL-6 and HLA-G (human leukocyte antigen). It has been determined that these mediators regulate the function and proliferation of various immune cells and the stimulation of TREG (regulatory T) cells directly or indirectly through the creation of immature dendritic cells (DCs) [40]. Along with the release of immunomodulators, MSCs can directly inhibit immune cell activation through cell-to-cell interaction. Direct contact between MSCs and T cells leads to inhibition of cell proliferation through induction of effector T cell apoptosis as the programmed death-1 (PD-1) molecules interact with its PD-L1 and PD-L2 ligands. Moreover, T cell anergy can be induced by MSCs through inhibition of the CD86 and CD80 expression in antigen-presenting cells [41–43].

MSCs also secrete various modulatory factors that are able to regulate inflammation, angiogenesis, cell death, tissue regeneration and fibrosis [44]. It was reported that they secrete trophic parameters that can increase cell survival, cell proliferation and tissue angiogenesis [45–47]. Furthermore, they are able to migrate toward injured tissues along chemoattractant gradients within the peripheral blood and stromal extracellular matrix (ECM) [48]. In injured tissues, local factors, such as cytokine milieu, hypoxia and Toll-like receptor ligands, stimulate MSCs, promoting the creation of growth factors in large quantity to drive tissue regeneration [49, 50].

On the other hand, studies have shown that MSCs can increase or decrease tumorigenesis under various conditions [51, 52]. Within its microenvironment, the tumor tries to prevent identification by the immune system, which establishes a stable inflammatory state by secreting inflammatory mediators [53]. There is an increasing focus on the interaction between cancer cells, normal cells and the matrix in the tumor microenvironment, because these interactions contribute to the milestones of cancer development, such as angiogenesis, immunomodulation, metastasis and invasion, as well as apoptotic resistance [54, 55]. In some research, it was indicated that MSCs travel to the cancer microenvironment, where they support the creation of the tumor vascular system and overwhelm immune reactions, thus modulating the tumor response to anti-tumor therapy [56–60]. MSC survival rates were also found to increase under oxidative, heat shock, hypoxic and nutrient-deprived conditions, which can occur in solid tumors, by inducing the expression of cytoprotective genes and promoting MSC potency through improving production and secretion of compensating factors [61–64]. Indeed, MSCs utilize an autophagic metabolism to provide the required nutrients for long-term survival and by secretion of survival factors helps to increase the survival rate of MSCs and the surrounding cells of them [65]. Contrary to their tumor-promoting capabilities, MSCs are also able to restrict tumor growth by suppressing angiogenesis, inhibiting proliferation-related signaling paths like PI3K, Wnt and AKT, and inhibiting cell cycle progression [66–71].

This review focuses on the supportive and suppressive impacts of MSCs on tumor progression and metastasis. We also discuss MSC-based therapeutic strategies for cancer, which are based on their potential for homing to tumor sites.

## The supportive effects of mesenchymal stem cells on tumor growth

MSCs display a robust tropism to damaged tissue and wounds, where they promote regenerative activities [50, 72]. In general, tumors, which can be compared to chronic non-healing wounds, also recruit MSCs to supporting their metastasis and growth [73–75]. The mechanisms of MSC homing is likely due to receptors and chemokines promoting the transfer of other accessory cells to the tumors. These would include growth factors (PDGF, SCF, HGF, IGF-1E and GF) [76, 77]; angiogenic factors (βFGF, HIF1α and VEGF) [78, 79]; chemokines (CCL5, CCL2, CXCL12 and CCL22) [76, 80]; cytokines and inflammatory factors (TGFβ, TNFα, IL-8 and IL-1β) [81–84]. MSC recruitment to various types of tumor may crucially contribute to the tumor fate. The capability of MSCs to promote metastasis and tumor growth was shown in a breast tumor model in mice [85]. Similar findings have been reported for MSCs co-implanted with cancer cells [86–88]. Moreover, tumor formation is not supported by findings for allogeneic mice with transplanted B16 melanoma cells but no concomitant MSC co-injection [89]. The supportive mechanisms of mesenchymal stem cell on tumor growth are presented in Table 1.

**Table 1:** The effect of MSC-derived mediators on tumor growth

Pro-tumorigenic activity of MSCs	Factors secreted by	Effects of secreted factors and mediators	References
Immunosuppression	*MSCs*: TGFβ, IFNγ, TNFα, PGE2, CCL2, galectin-9, HGF, CTLA-4, soluble PD-L1 and PD-L2, NO, HLA-G, IDO, IL-1α, IL-1β, IL-4 and IL-6	Immune tolerance T, B, NK, Dendritic cell inhibition Promotion of Treg cells proliferation Recruitment of MDSCs Apoptosis of lymphocytes and neutrophils Reduction of CD80/CD86 expression on APCs	[40, 92–96, 103–105, 109]
Promotion of angiogenesis	VEGF, FGF-2, βFGF, PDGF, IL-6, IL-8, TGFβ and angiopoietin-1	Promotion of tumor angiogenesis Transformation into smooth muscle cells and pericytes Mobilization and recruitment of MSCs into neovascularization sites Tumor vessel formation Inducing expressing of junctional proteins	[51, 88, 123, 124, 127–129, 135–137]
Transition of mesenchymal stem cells to cancer-associated fibroblasts	*CAFs*: α-SMA, tenascin-C, fibroblast surface protein (FSP), CCL5, CXCL12, IL-6, IL-4, IL-8, TNF, TGFβ, VEGF	Stimulation of tumor growth Promotion of tumor vascularization	[88, 142–144]
Epithelial–mesenchymal transition (EMT)	HGF, EGF, PDGF, leptin and TGFβ	Induction of transcriptional regulators: snail, slug, twist, Zeb1 Increasing the metastatic capacity Inducing EMT and promoting a cancer stem cell (CSC) phenotype	[152–154, 157, 158]
Correlation of MSCs with cancer stem cells	BMP, IL-6, IL-8, CXCL6, and CXCL5	Proliferation of CSCs and increasing their invasive properties	[90, 164, 165]
Promotion of tumor metastasis	Lysyl oxidase (LOX), TGFβ, FGF, HGF, EGF, CCL5, CXCL5, CXCL1, CXCL7 and CXCL8	Promotion of tumor cell migration Extracellular matrix modulation Enhancing tumor cell invasiveness and inducing EMT Activation of matrix metalloproteinase 9 (MMP-9) Overexpression of rho-associated kinase	[59, 75, 167, 171, 173–175]
Inhibition of apoptosis in cancer cells	VEGF, FGF-2, PDGF, HGF, BDNF, SDF-1α, IGF-1 and IGF-2, TGF-β and IGFBP-2	Inhibition of tumor cell apoptosis and promotion of tumor proliferation Stimulation of the angiogenesis	[52, 184–188]
Promotion of drug resistance	CXCL12, EGF, IGF, IL-6, IL-7, IL-8 and PGE-2	Reducing caspase 3 activity Inhibition of apoptosis following cytotoxic therapy Promotion of the CSCs formation	[91, 133, 164, 196, 198]

### Suppression of the immune response in tumor microenvironments

**Fig. 1:**
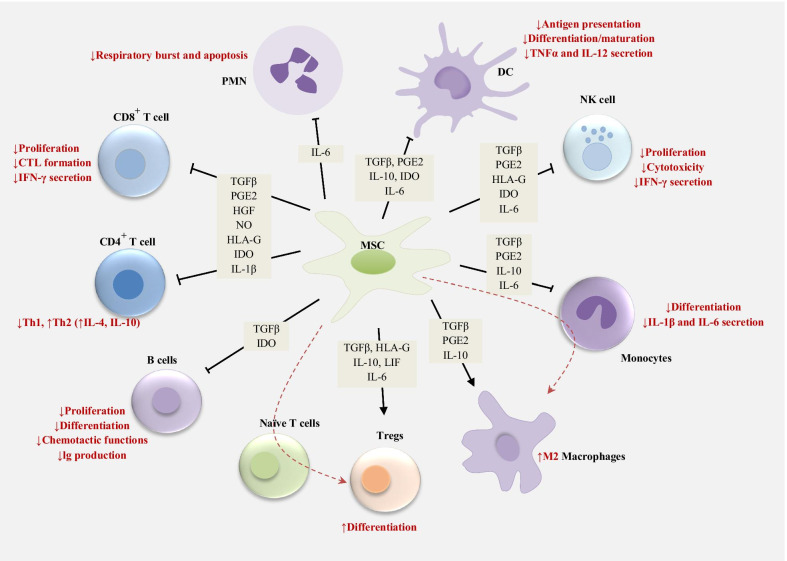
MSC-mediated immunosuppression

MSCs have robust immunosuppressive features that support the tumor cells to escape from immune surveillance (Fig. 1). MSCs can be activated within tumor microenvironments by the pro-inflammatory cytokines TNF-α, IFN-γ or IL-1β [82, 90–92], which are secreted by macrophages and tumor cells. Extensive immunosuppression is then induced, mainly by MSCs through the secretion of mediators and soluble factors, such as indoleamine 2,3-dioxygenase (IDO), TGFβ, TNFα, IFNγ, prostaglandin E2 (PGE2), NO, HLA-G, HGF, IL-1β, IL-1α, IL-4 and IL-6, and through their interactions with different types of immune cell, including B cells, T cells, dendritic cells, NK cells and macrophages [92–94]. Considering the expression of both HLA-G and IDO in the placenta, MSCs probably contribute to immune tolerance during pregnancy. These mediators reduce effector T cell proliferation, IgG secretion, B cell proliferation, DC maturation, and nature killer (NK) cell activity [95, 96]. IDO is involved in the maturation of T helper cells into FOXP3-positive regulatory T cells that can inhibit effector T cell responses and thus reduce anti-tumor immunity [97, 98]. PGE2 induces secretion of anti-inflammatory cytokines like IL-10 and reduces the expression and synthesis of IFN-γ, TNF-α and IL-12 in macrophages and dendritic cells (DCs) [99]. PGE2 also diminishes the secretion of IL-4 and IFN-γ in Th2 and Th1 cells, respectively, while promoting the proliferation of Treg cells [40]. Besides, MSCs induce skewing of the pro-inflammatory Th1 CD4 cells into an anti-inflammatory Th2 phenotype [100, 101]. Reducing the IFNγ production by T Helper 1 and enhancing IL-4 secretion via T Helper 2 minimizes anti-tumor immunity and the immune response. Monocyte differentiation is inhibited by MSC-secreted IL-6 toward DCs, reducing the capability of DCs to stimumate T cells [102, 103]. Furthermore, MSC-secreted IL-6 led to the delay in apoptosis of neutrophils and lymphocytes [104, 105]. NO is synthesized by inducible NO synthase (iNOS) through stimulation by inflammatory factors, such as IFN-γ, IL-1, and TNF-α [106, 107] and then inhibition of the T cell functions [108].

Ultimately, recruitment of the inhibitory immune cells known as MDSCs (myeloid-derived suppressor cells), is induced by MSCs through CCL2 signaling. This weakens the anti-cancer T cell activity [109]. Ucelli et al. found that MSCs prevent the proliferation of B cells by arresting the cell cycle [15]. B cell antibody production is reduced by MSCs, but this also inhibits their differentiation into plasma cells [110]. Galectin-9 is overexpressed by IFNγ-stimulated MSCs, attenuating antibody production and B cell proliferation [111]. Thus, MSCs show a robust inhibitory performance against adaptive immune cells and this capability is exploited by cancer cells.

NK cells are also suppressed by MSCs, mainly through IL-6 and PGE2 secretion. Furthermore, MSCs decrease NK cells’ ability to express IFNγ, weakening their anti-cancer activity [112]. Moreover, MSCs attenuate the maturation of dendritic and other antigen-presenting cells (APCs) through PGE2 signaling [99]. Macrophage phagocytic capabilities are reduced when exposed to a conditioned medium of MSCs, thereby stimulating a pro-tumorigenic macrophage phenotype [113]. CD80/CD86 expression in APCs is reduced by MSCs, thus downregulating T cell activation [103]. Furthermore, MSC-derived PGE2 and IDO induce the skewing of macrophages toward the pro-tumorigenic M2 phenotype, leading to the higher immune-inhibitory IL-10 levels [114]. Moreover, co-culturing CD11b/Ly6G-positive neutrophils with MSCs leads to massive in vitro inhibition of T cells, which was found to increase the growth of breast carcinoma in vivo [115]. There are also other reports indicating that neutrophil activation is promoted in gastric cancer by MSC-derived IL-6 through the STAT3-ERK1/2 signal transduction pathway and that their polarization is induced toward a tumor-supportive phenotype [116]. These findings represent the MSCs’ extensive immunosuppressive impact on effector immune cells, which ultimately supports the progression of the tumor.

The high IFN-γ levels within the pro-inflammatory intra-tumor environment increase the expression of PDL1 on MSCs, in turn inhibiting T cell activation and attenuating the activity of anti-cancer T cells [117]. Furthermore, high levels of soluble PD-1 ligands are secreted by MSCs, effectively suppressing the impact of IL-2 on T cell activation. In parallel, AKT activation paths are inhibited by the soluble PD-L1 and PD-L2 secreted by MSCs, so T cell activation and proliferation are inhibited [118]. Besides the T cell inhibition that is mediated by PD-1 and PD-L1, it was recently found that MSCs’ immunosuppressive performance is partly mediated through CTLA-4 that MSCs express several types of them [119].

### Angiogenesis promotion

Fast maturation and expansion of the tumor vasculature is essential to satisfy the high requirement for nutrients and oxygen in primary metastatic sites and tumors [120]. Various studies indicate that tumor angiogenesis is promoted by MSCs that differentiate into pericytes or endothelial-like cells and then secrete proangiogenic factors, trophic factors, cytokines, plasminogen activators and growth factors [88, 121, 122]. MSCs release high levels of cytokines and angiogenesis-stimulating growth factors, including VEGF, βFGF, FGF-2, PDGF, IL-8, IL-6, angiopoietin and TGFβ, which promote tumor angiogenesis [88, 123, 124]. Moreover, MSC-derived VEGF production is enhanced by high TGFα levels by inducing PI3K and MAPK paths, in turn activating the paracrine and autocrine loops and stimulating pro-angiogenic factor secretion [125, 126]. VEGF expression can increase the recruitment and mobilization of MSCs into neovascularization sites, where MSCs are directly differentiated into vascular cells [51, 127]. Furthermore, MSCs secrete cytokines like angiopoietin-1 (Ang-1) and IL-6 in colorectal carcinoma, in turn stimulating cancer cells to secrete endothelin-1 (ET-1), which in turn activates ERK and AKT paths in tumor endothelial cells, resulting in tumor vessel formation and mobilization [128, 129]. The secretion of VEGF by MSCs can be incremented by hypoxic conditions as the prevalent event in tumor tissues [130]. Hypoxia results in HIF-1α overexpression downregulating p21 and p53 and upregulating BCL-2 in MSCs, thus, promoting their survival [131]. Pro-angiogenic factors, such as PDGF and VEGF, are secreted by these surviving MSCs, resulting in local tumor angiogenesis [132, 133]. Furthermore, endothelial cell recruitment is stimulated by VEGF secretion from MSCs in a hypoxic environment, supporting systemic and local tumor angiogenesis [134]. Moreover, blood vessel integrity is enhanced by MSCs through the induction of junctional protein expression [135]. The expression of cell-to-cell junction proteins, such as occludin is enhanced by MSC-derived Ang-1 in endothelial cells, reducing blood vessel leakiness [135]. The angiogenic procedure is also directly enhanced by MSCs that have differentiated into endothelial cells, pericytes and smooth muscle cells [136]. It was reported that MSCs are capable of differentiating into smooth muscle cells, and therefore can support tumor blood vessel maturation [137]. Indeed, differentiated pericytes supporting blood vessel integrity are regarded as originating from MSCs [138, 139]. Endothelial markers were not expressed by rat MSCs incorporated into tumor vessel walls including NG2, PDGFR-β, and α-SMA [122]. The various pericyte marker expression profiles of engrafted MSCs and their perivascular position indicates the function of these cells as pericytes. Numerous specific markers for pericyte differentiation are expressed in human MSCs cultured in conditioned media derived from a glioblastoma [140, 141]. Owing to the MSCs’ pro-angiogenic nature into the growing tumors, current investigations are focusing on blocking the MSCs’ angiogenic activities to enhance the anti-angiogenic therapy.

### Conversion of MSCs to cancer-associated fibroblasts

Kalluri et al. indicated that MSCs are resting fibroblasts and cancer-associated fibroblasts (CAFs) can be derived from them [142]. MSC–tumor cell interactions further augment MSC differentiation into CAFs [88, 143, 144]. CAFs are abundant in most invasive tumors and mostly include cells expressing *α*-SMA (*α*-smooth muscle actin) [145]. They promote angiogenesis and tumor growth through the secretion of SDF-1 (stromal-cell derived factor 1) [146], which binds to their receptor, CXCR4 [147]. Fibroblast-derived mediators and factors like α-SMA, tenascin-C, fibroblast surface protein (FSP), CXCL12, IL-6, and CCL5 are extensively produced by MSCs [88]. Tumor growth can be stimulated by CAFs and TAFs through their involvement in angiogenesis and the secretion of tumor-stimulating growth factors. Tumor angiogenesis is strongly promoted by CAFs through the production of immunomodulatory and pro-angiogenic chemokines and cytokines like IL-4, IL-8, IL-6, TNF, CXCL12, TGFβ and VEGF [142]. Therefore, tumor vascularization is promoted by MSCs through multiple mechanisms related to induction of vasculogenic mimicry and endothelial cell proliferation.

### Epithelial–mesenchymal transition

Epithelial–mesenchymal transition (EMT) is determined by downregulation of the epithelial cell-related proteins E-cadherin, ZO-1 and γ-catenin/plakoglobin. However, the mesenchymal proteins are upregulated, such as fibronectin, N-cadherin, smooth muscle actin and vimentin [148, 149]. Although EMT is needed for wound healing and organogenesis, it is also related to epithelial tumor development [150]. Evidence indicates that tumor invasiveness, drug resistance and tumor metastasis are promoted by aberrant EMT [151]. EMT is promoted by MSCs through the secretion of cytokines and growth factors, including HGF, PDGF, EGF and TGFβ, which induce the expression of transcriptional regulators related to EMT, such as Slug, Snail, Zeb1 and Twist [152, 153]. MSCs improve colon cancer cells’ metastatic capacity through the increased expression of EMT-related genes, such as ZEB2, ZEB1, Twist, Slug, and Snail, in a contact-reliant mode. The EMT-associated gene E-cadherin is considerably downregulated in this process [154]. In hepatocellular carcinoma, the exposure of MSCs to IFNγ and TNFα leads to overexpression of TGFβ, which in turn promotes EMT-associated functional alterations in cancer cells [155]. A metastatic phenotype is promoted by TGFβ-stimulated MSCs in pancreatic cancer via the upregulation of Jagged-1, a key ligand of Notch signaling in tumor cells [156]. In turn, EMT is induced by activation of the Notch path increase a cancer stem cell (CSC) phenotype. Other works linking EMT with CSCs support these results [157]. The expressions of metastatic and EMT genes (SERPINE1, IL-6, and MMP-2) were induced in tumor cells by MSCs secreted leptin. Moreover, when co-injecting MCF7 breast cancer cells and transfected MSCs with leptin shRNA into SCID/beige mice, a reduction in leptin levels in MSCs occurs, finally causing a decrease in the number of metastatic lesions and in the MCF7 tumor volume in the mouse livers and lungs [158]. There are also reports on another mechanism of tumor cell dissemination induced by MSCs in gastric cancer. MSCs recruited by gastric mucosal cells infected with *Helicobacter pylori* differentiated into gastric cells expressing epithelial biomarkers like TFF2 and KRT1-19. The gastric cancer CSC phenotype is promoted by such biomarkers accompanied by chronic inflammation contributing to metastatic and EMT features [159]. Furthermore, MSCs can also fuse with various tumor cells that represent all the classical features of EMT [158, 160–162].

### Correlating MSCs with CSCs

Some evidence indicates that tumor metastasis is mediated by CSCs, which are probably involved in relapses following radiation therapy and chemotherapy [163]. Liu et al. found that a cellular hierarchy is formed by MSC and CSC populations where breast CSCs are regulated by MSCs expressing aldehyde dehydrogenase through cytokine loops including CXCL 7 and IL-6 [164]. When an interaction occurs between IL-6 secreted by CSCs and IL-6R/gp130 expressed on MSCs, MSCs express CXCL7 [164]. In turn, the secretion of some cytokines, including IL-8, IL-6, CXCL5, and CXCL6, from both MSCs and CSCs is induced by CXCL7 [164]. It was indicated that the invasive properties and proliferation of CSCs are promoted by these cytokines, and that IL-6 promotes MSCs homing to the tumor sites in mouse xenograft models [90, 164]. BMP6, BMP4 and BMP2 are expressed by carcinoma-associated MSCs (CA-MSCs). In vitro culture of CSCs with BMP2 mimicked the impact of CA-MSCs on CSCs, while in vivo and in vitro MSC-promoted tumor growth was partly suppressed after the BMP2 signaling pathway was inhibited. These findings reveal that MSCs can stimulate tumor progression by incrementing the cancer stem cell number via BMP expression [165].

### Tumor metastasis promotion

Several investigations demonstrated the influence of MSCs on cancer cell invasion and migration, EMT, and the creation of secondary metastatic lesions [75, 166–168]. These impacts are partly due to various growth factors, chemokines, and cytokines secreted by MSCs. C–X–C and C–C are among the chemokines that promote tumor cell migration to secondary sites [169–172]. Growth factors like TGFβ, HGF, FGF and EGF enhance tumor cell invasiveness and induce EMT [75, 173, 174] as well as extracellular matrix modulating factors like lysyl oxidase (LOX) [167, 175]. In breast carcinoma, CCL5 is secreted by tumor-residing MSCs [90]. There is also a close relationship between CCL5-mediated invasion and the incremented activity of matrix metalloproteinase 9 (MMP-9) [59]. Nevertheless, this improved metastatic capacity is reversed by injecting MSCs into separate places, even in close proximity [90]. In another study, it was indicated that breast cancer cell directional migration and elongation are induced by co-cultured MSCs in vitro. The TGFβ signaling pathway in MSCs mediates the overexpression of matrix metalloproteinases (MMPs), rho-associated kinase and focal adhesion kinase (FAK) in breast cancer cells [174]. E-cadherin is downregulated by MSCs in tumor cells through the activation of ADAM10 and inhibition of the epithelial-like tumor cell phenotype [176]. Therefore, this activity of MSC disrupts cell–cell contact, thus increasing cell migration capabilities and promoting metastasis [176, 177]. Tumor cell migration to metastatic lesions is promoted through MSC secretion of chemoattractants, such as CXCL1, CCL5, CXCL5, CXCL8 and CXCL7 [90, 174]. Furthermore, it was reported that tumor-derived osteopontin (OPN), a multipotent chemoattractant, promotes the expression of CCL5 in MSCs, which leads to incremented metastasis [86, 178]. Moreover, it was reported that high levels of CXCL12 (SDF-1) are secreted by MSCs that regulate the migration and invasion of CXCR4-expressing tumor cells [179, 180]. Moreover, bone metastasis can be supported by MSCs through the creation of metastatic niches at the bone and contribution to cancer cell recruitment to the bone ECM. These capabilities are related to their robust adhesive activities, which are facilitated by their secretion of integrins and adhesive molecules [181]. In general, MSCs contribute to the numerous stages within the metastatic procedure and have proven vital at pre-metastatic and primary tumor sites. Other mechanisms, such as the regulation of CSCs, induction of EMT, and changing mesenchymal niches also contribute to tumor invasion and metastasis [182].

### Inhibiting the apoptosis in cancer cells

Tumor progression is associated with hypoxia, inflammation and starvation. Culturing MSCs in vitro under hypoxic circumstances incremented cellular proliferation and enhanced the production of Oct-4 and Rex-1. Thus, an increase in MSC stemness during hypoxia was concluded [183]. Additionally, under starved and hypoxic circumstances, MSCs can increase their survival rate through autophagy and the secretion of numerous pro-survival or anti-apoptotic factors, including VEGF, PDGF, FGF-2, brain-derived neurotrophic factor (BDNF), SDF-1α, HGF, IGF-2, IGF-1, IGF binding protein-2 (IGFBP-2) and transforming growth factor-β (TGF-β) [184–188]. These factors can inhibit tumor cell apoptosis and promote tumor proliferation, these features are not seen in MSCs under normal conditions. In addition to the mitogenic features of growth factors released by MSCs, VEGF and FGF-2 can also induce the expression of Bcl-2, prolonging survival and delaying apoptosis [189–191]. However, the expressions of FGF-2 and VEGF can be induced by indirect angiogenic factors [192]. Moreover, it was reported that SDF-1α prevents chronic lymphocytic leukemia (CLL) cells’ drug-induced apoptosis [193]. It was also indicated that the angiogenic and anti-apoptotic impacts of hypoxic conditioning are stimulated by MSCs-derived factors, such as VEGF, HGF, FGF-2 and IGF-1 [49, 187]. Although less information exists about the direct supportive impact of MSCs on tumor cells under hypoxic circumstances, we know about the supportive potential of MSC-secreted growth factors triggered by hypoxia in the tumor microenvironment from observations of their angiogenic and anti-apoptotic impact.

### MSCs promote drug resistance

Cancer chemoresistance mechanisms are not only related to aberrant gene mutations and multidrug resistance proteins, but also to different factors and conditions within the tumor microenvironment [194]. Numerous studies indicated that MSC pro-tumorigenic activities are improved in response to tumor perturbation, explaining chemoresistance and cancer regrowth [73]. In chronic myeloid leukemia (CML), CXCL12 is secreted by MSCs, reducing caspase 3 activity in a CXCR4-dependent manner and attenuating cell death induced by imatinib [133]. It was reported that in chronic lymphoid leukemia (CLL), CLL cells are protected from the cytotoxic impacts of Forodesine by MSCs enhancing the RNA and protein expression in the tumor cells [195]. It was also revealed that MSCs increase resistance to treatment in solid tumors. For example, in neck and head carcinoma, chemoresistance to paclitaxel is induced by bone marrow-derived mesenchymal stem cells (BM-MSCs) through paracrine signaling of IGF, EGF, IL-6, IL-7 and IL-8 [196]. It was found that in ovarian cancer, cancer cells are protected from cell death-induced hyperthermic intraperitoneal chemotherapy by tumor-residing MSCs that activate the CXCL12–CXCR4 axis. The thermo-sensitivity of ovarian cancer cells can be restored through blockage of the CXCR4 axis [197]. IL-6 expression by MSCs is another mechanism supporting tumor resistance. IL-6 stimulates Bcl-2 and Bcl-XL synthesis in cancer cells, resulting in the prevention of apoptosis after cytotoxic therapy [198]. Polyunsaturated fatty acids are secreted by MSCs that are exposed to cisplatin, protecting tumor cells from drug cytotoxicity. However, the mechanism of this protection is not known [199]. That study also showed that chemoresistance can be induced by non-tumor-residing MSCs and that MSCs can be considered guarding cells that protect cells from cytotoxic agents.

Previously, we discussed the involvement of MSCs in metastasis through facilitating EMT. Cancer cells that have undergone EMT are regarded as cancer stem cells or tumor cells with CSC properties [157, 200]. The CSCs are an exclusive rare population of cells within tumors. They show resistance to numerous cytotoxic agents, partly owing to their low proliferative rate, high DNA repair mechanisms, and expression of membrane drug transporters [201, 202]. Here, we discuss the capability of MSCs to either transform into CSCs or to support their microenvironment. MSCs can differentiate into CSCs by specific methylation in the tumor suppressor genes, HIC1, and RasF1A. Then, CSCs increase the chemoresistance to cisplatin and the risk of tumor relapse followed by treatment cessation [203]. Simultaneously, a CSC niche is supported by MSCs, and this contributes to their capability to resist chemotherapy toxicity [204, 205]. In breast cancer, CSCs are regulated by MSCs via cytokines like CXCL-7 and IL-6, thus promoting cancer progression[164]. In colorectal cancer, IL-1 released by cancer cells leads to the secretion of PGE-2 by MSCs. Through autocrine effects, that PGE-2 induces the production of IL-8, IL-6 and CXCL1, and these factors promote the formation of CSCs [91]. After differentiating into CAFs, MSCs are able to preserve CSCs by secreting the Notch ligand Jagged-1 [156]. Moreover, cisplatin changes the phosphorylation of various tyrosine kinase enzymes in MSCs, such as c-Jun, WNK-1, STAT3 and p53 that these proteins increase MSC survival and synthesis of IL-8 and IL-6 by MSCs, enhancing tumor cell chemoresistance [206]. However, the exact mechanisms of reduced chemoresistance by MSCs have not been fully clarified.

It was indicated that a physiological response to chemotherapy agents occurs in MSCs and eventually decreases chemoresistance by increasing the CSC population. MSCs are recruited numerously to pancreatic cancer sites in response to gemcitabine therapy, and they localize close to the CSC niches to support them. CXCL10 synthesis significantly increases in gemcitabine-exposed MSCs and promotes the proliferation of CSCs that have surface overexpression of CXCR3, which is a CXCL10 receptor. Ultimately, all these events lead to increased chemoresistance and enhanced tumor growth [207].

## Suppressive impacts of MSCs on tumor growth

Although the tumor-promoting features of MSCs are clear from several studies, numerous other studies indicated tumor-suppressive features [56]. It is thought that MSCs suppress tumor growth by inhibition of Wnt and AKT signaling [66–68], suppression of angiogenesis [71], promotion of inflammatory cell infiltration [208], and induction of cell cycle arrest and apoptosis [70, 193, 209, 210]. It has been shown that IFN-β is produced by adipose tissue-derived MSCs cultured at high cell density, which reduces MCF-7 cell growth [211]. Furthermore, TRAIL is expressed by MSCs cultured with tri-dimensional systems or primed with IFN-γ, which can increase cancer cell-specific apoptosis [212, 213].

### Regulating the cell cycle

The expressions of cyclin A, cyclin D2, p27KIP1, and cyclin E can be induced by cytokines that are secreted by MSCs. Their action ultimately leads to the cell cycle arrest of cancer cells at the G1 phase [204, 210, 214, 215]. It was found that human stromal cells differentiated from adipose tissue (ADSC) and ADSC-conditioned cell culture medium can suppress tumor progression [210]. Furthermore, in the absence of apoptosis, ADSC-conditioned cell culture medium promotes cancer cell necrosis after G1-phase arrest. Tumor growth was inhibited when ADSC infiltrates pancreatic adenocarcinoma [210]. Similarly, the co-culture of cancer cells with MSCs also leads to cell cycle arrest of tumor cells at the G1 phase [204]. Nevertheless, co-injection of cancer cells with MSCs into non-obese, diabetic-severe, combined immunodeficient mice yielded higher tumor cell growth than tumor cell injection alone. The exact mechanisms of cell cycle arrest of tumor cells by MSCs remain unknown.

### Inducing inflammatory infiltrates

Although immune responses can be suppressed by MSCs, in vivo co-administration of cancer cells and MSCs led to the enhanced infiltration of granulocytes and monocytes compared to separate cancer cell or MSC injection. In the study of Ohlsson et al., a gelatin matrix containing rat colon cancer cells and/or MSCs was utilized and transplanted subcutaneously into rats to monitor outgrowth of the tumor and the resultant inflammatory response. Rat colon carcinoma was inhibited by MSCs [208]. Infiltrations of macrophages and granulocytes were much greater in rats co-injected with MSCs and cancer cells than in rats injected with cancer cells without MSCs. These findings indicate the pro-inflammatory properties of MSCs in this model. However, the expressions of MHC-class I and MHC-type II in MSCs were low or absent respectively. Indeed, studies revealed a much smaller enhancement of granulocyte and macrophage infiltration when MSCs alone were added to the gelatin [208].

### Stimulation of apoptosis of tumor cells and endothelial cells

The suppressive impacts of MSCs on tumor cell growth have been shown in the absence of host immunosuppression conditions, by inducing cancer cells apoptosis and G0/G1 cell cycle arrest. MSCs were found to promote p21 expression [210]. Furthermore, MSCs were found to have anti-cancer activity in SCID mice xenografted with disseminated non-Hodgkin’s lymphoma [216]. A single injection of MSCs enhanced the survival of animals with high-grade lymphoma. A considerable stimulation of endothelial cell apoptosis was indicated in a direct co-culture of endothelial cells with MSCs, indicating that they can have the anti-angiogenic activity [216]. The results concurred with some other studies that demonstrate a robust anti-angiogenic property of MSCs in Kaposi’s sarcomas with high vascularity [66, 71]. Additionally, it was reported that human umbilical cord blood-derived MSCs can induce the apoptosis of xenograft cells and glioma cells through downregulation of the X-linked inhibitor of apoptosis protein (XIAP) by activating caspase 3 and caspase-9 [70, 209]. High-density cultures of MSCs were found leading to the overexpression of type I IFN, which causes cell death of MDR-MB-231, MCF-7 and breast cancer cells [211]. Furthermore, TRAIL can be expressed by MSCs cultured with tri-dimensional systems or primed with IFN-γ that increases cancer cell apoptosis [211, 213].

### Regulation of cellular signaling

Cell growth, migration, survival, metabolism and proliferation are controlled by WNT/β-catenin and PI3K/AKT signal transduction pathways [217–222]. Numerous studies have shown the importance of AKT signaling for the survival, invasion and migration of tumor cells. The Wnt signaling pathway has also been involved in the development of the breast, colon, liver, ovary, stomach and skin cancer [223–231]. It was reported that intravenously injected MSCs can travel to tumor sites and significantly decrease tumor cell proliferation by inhibiting the AKT signaling pathway in a Kaposi’s sarcoma model [66]. Also, PTEN was upregulated in glioma cells by human cord blood-derived stromal cells (hUCBDSCs) leading to the downregulation of AKT [209]. Furthermore, MSCs are able to inhibit the WNT/β-catenin signaling path by the induced expression of DKK-1 [67–69]. The results show the downregulation of β-catenin by DKK-1 secreted from MSCs in human carcinoma cell lines (breast: MCF-7; hepatocellular: HepG2 and H7402; and hematopoietic: HL60 and K562). Inhibiting DKK-1 activity by utilizing RNAi or neutralizing anti-DKK-1 antibodies results in reduced inhibitory impacts of MSCs on tumor cell proliferation [67–69].

## The therapeutic potential of engineered MSCs as a Trojan horse in cancer

The wide application of anti-cancer and anti-proliferation agents is restricted by their high toxicity or short biological half-life. Cell-based therapy has recently emerged as one of the solutions to these limitations. Because of their inherent migratory properties, MSCs have attracted considerable attention among different cell types. These properties could allow them to be used as delivery vectors for anti-cancer therapy [232, 233].

As mentioned throughout this paper, the exact function of MSCs in cancer settings is still debatable and controversial. To solve the problem of their duality effect on cancer cells, researchers are trying to engineer MSCs to convert them into indisputable therapeutic tools and serve as an anti-cancer Trojan horse [234]. It is possible to equip MSCs with various factors. They have typically been genetically engineered to express desirable cytokines, anti-angiogenic, pro-apoptotic, anti-proliferative agents that specifically target different cancers, but have also been used as carriers for anti-cancer drugs [234]. Furthermore, both tumor-promoting and tumor-suppressive behaviors have been observed for MSC-derived microRNA [235–237]. Thus, MSCs can also be engineered to express desired and specific anti-cancer miRNA.

Several studies utilized genetically manipulated MSCs to deliver and express various anti-tumor agents, including type I interferon (IFN-α and IFN-β), CXCL1, IL-2, IL-12, cytokine deaminase, oncolytic virus, TRAIL and nanoparticles [238–249]. Alternatively, engineered MSCs can express particular enzymes, such as herpes simplex virus-thymidine kinase (HSV-TK) or cytosine deaminase, which can convert inactive systemically administrated prodrugs, such as ganciclovir and fluorouracil (5-FU), into active cytotoxic drugs, reducing potential and systemic toxicity by facilitating tumor-localized chemotherapeutic activity [250, 251]. MSCs are utilized in prostate cancer to promote drug specificity by delivering inactive prodrugs that can be activated in the cancer site through tumor-specific enzymes, including prostate-specific membrane antigen (PSMA) or prostate-specific antigen (PSA) [252, 253]. Moreover, MSCs can be utilized for effective targeting of nanoparticle-based drug delivery systems [247]. Taken together, these various approaches show the power of MSCs as effective anti-cancer delivery systems that are defined by reduced systemic toxicity and enhanced cancer specificity. The immune response is improved against glioma by IL-2-overexpressing MSCs [245] and melanoma [254] and decrease metastasis and invasion from a subcutaneous models [255]. NK and T cells are activated by IL-12 and CXCL1 secreted by MSCs, leading to a substantial decrease in breast and lung tumors and melanoma [244, 246, 255–257]. Since genetically engineered MSCs can increase the local concentrations of TRAIL, IFN-α and IFN-β, their tumor-suppressive activities are more effective compared to their activities when utilized in a systematical therapy [238, 240, 242, 243]. It was also found that CSCs can be specifically targeted by TRAIL-expressing MSCs in lung carcinoma, reducing chemoresistance, tumor aggressiveness and relapse rates [239]. MSCs have also been modified to deliver conditional replicative oncolytic viruses for specific targeting and inhibition of cancer cells without impact on normal cells, thus reducing metastasis and tumor growth [241, 258, 259]. In general, genetically modified MSCs are considered a promising therapeutic tool.

MSC therapy has numerous potential drawbacks that probably limit the expected clinical benefit, including the non-effective local concentration of therapeutic agents within the cancer microenvironment and non-specific distribution throughout the organism [260]. Furthermore, their physiological differentiation into mesenchymal lineages may promote immunogenicity, induce tumorigenesis and reduce therapeutic potential [261]. To overcome these challenges, MSC-derived extracellular vesicles (EV) were prepared and used as a drug delivery vector to directly target the cancer cells. The tumor homing ability is maintained by these MSC-derived EVs [262] with similar immune-suppressive features to the original MSCs [263]. In this concern, it was found that miRNA, cytokines, proteins and adhesion molecules can be delivered by exosomes from BM-derived MSCs and thereby influencing cancer development. For instance, glioma tumor growth was reduced by injecting exosomes obtained from miR-146b expressing MSCs [264]. Moreover, glioma stem cell survival was decreased by MSC-derived exosomes expressing miR-124a, and the migration properties of osteosarcoma cells were reduced by miR-143-containing exosomes [265, 266]. It was also reported that genes driving tumorigenesis can be silenced by MSC-derived EVs loaded with siRNA [267]. It is also possible to load MSC-derived EVs with cytotoxic chemotherapy agents, such as doxorubicin, paclitaxel or gemcitabine. Such EVs were found to suppress oral squamous cancer cell growth and decrease cell viability [268].

Nanovesicles formed from MSC-derived nanoghosts (NGs; i.e., the outer membrane of mesenchymal stem cells) provide another type of MSC-derived EVs that display the unique features of MSCs [269]. MSC adhesion receptors and molecules are mainly preserved in NGs, so they maintain the capability to home to tumors [207, 270]. It was reported that lung and prostate tumor growth is inhibited by NGs loaded with RNA [270]. Moreover, CXCR3 antagonist containing MSC-derived NGs increase the sensitivity of pancreatic tumor CSCs to therapy, enhancing chemotherapy efficacy and postponing cancer re-growth [207]. In general, the immunosuppressive and regenerative properties of MSCs represent their potential as therapeutic tools for different kinds of malignancy.

## Conclusions and perspective

**Fig. 2:**
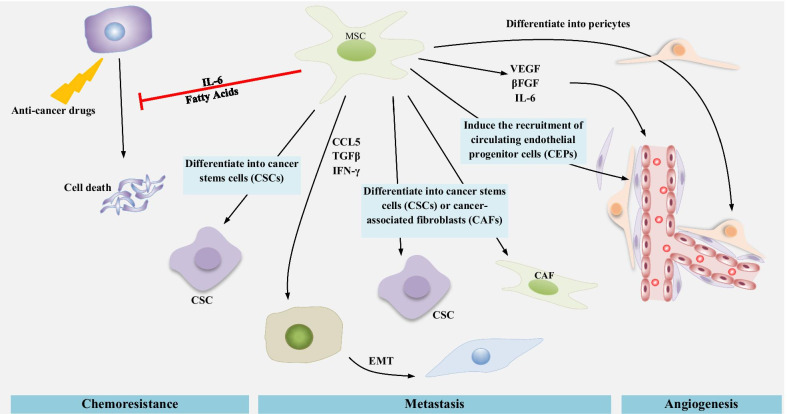
MSCs induce tumor-supportive processes including chemoresistance, metastasis and angiogenesis

MSCs are still one of the most promising therapeutic tools in tissue engineering and regeneration, cancer, and wound healing. Different cell types can affect tumor growth. Tumor cells and MSCs interact in various ways, hence, the MSCs either suppressing or supporting cancer progression based on multiple factors. MSCs support tumor growth through various mechanisms, including promotion of drug resistance, pro-angiogenic function, promotion of metastasis through induction of EMT, CSC niche enrichment, and profound immunoinhibitory properties (Fig. 2). These activities are related to mesenchymal stem cells secreted factors that affect several hallmarks of cancer. Moreover, the timing of MSC entrance into the tumor site may be vital for determining how they impact tumor development.

The homing ability of MSCs makes them excellent candidates as drug delivery vectors for therapeutic agents. More studies on the tumor growth mechanisms of MSCs can increase the potential to use them in regenerative medicine through improvements in their preparation to ensure minimal side effects and little or no risk of tumor cell growth. To further develop current anti-cancer treatment strategies, a better comprehension of the specific molecular mechanisms underlying these pro-tumorigenic activities is critical. Attenuation of MSC recruitment into tumor sites and inhibition of their tumor-supportive activities will make it possible to enhance the therapeutic outcomes for cancer patients, especially with the integration of other anticancer approaches, such as immunotherapy. Genetically modified MSCs possess great potential to specifically target tumor cells with minimal adverse effects and systemic toxicity.

## Data Availability

Not applicable.

## Abbreviations

*α*-SMA: *α*-Smooth muscle actin
Ang-1: Angiopoietin-1
APC: Antigen-presenting cell
BDNF: Brain-derived neurotrophic factor
CAF: Cancer-related fibroblast
CA-MSC: Carcinoma-associated MSCs
CLL: Chronic lymphocytic leukemia
CML: Chronic myeloid leukemia
CSC: Cancer stem cell
DC: Dendritic cell
ECM: Extracellular matrix
EMT: Epithelial–mesenchymal transition
ET-1: Endothelin-1
EV: Extracellular vesicle
FSP: Fibroblast surface protein
HLA: Human leukocyte antigen
HSV-TK: Herpes simplex virus-thymidine kinase
IDO: Indoleamine 2,3-dioxygenase
IGFBP-2: IGF binding protein-2
iNOS: Inducible NO synthase
ISCT: International society for cellular therapy
LOX: Lysyl oxidase
MDSC: Myeloid-derived suppressor cell
MMP-9: Matrix metalloproteinase 9
MSC: Mesenchymal stem cell
NK: Nature killer
NO: Nitric oxide
PD-1: Programmed death-1
PGE2: Prostaglandin 2
PI3K: Phosphoinositide 3-kinase
PSA: Prostate-specific antigen
PSMA: Prostate-specific membrane antigen
TGF-β: Transforming growth factor-β
XIAP: X-linked inhibitor of apoptosis protein
